# Microarray Expression Profiles of lncRNAs and mRNAs in Postoperative Cognitive Dysfunction

**DOI:** 10.3389/fnins.2018.00694

**Published:** 2018-10-08

**Authors:** Ying Zhang, Yue-Xin Liu, Qiu-Xia Xiao, Qing Liu, Rui Deng, Jiang Bian, Isaac Bul Deng, Mohammed Al-Hawwas, Feng-Xu Yu

**Affiliations:** ^1^Department of Anesthesiology, Affiliated Traditional Chinese Medicine Hospital, Southwest Medical University, Luzhou, China; ^2^School of Pharmacy and Medical Sciences, Sansom Institute, Division of Health Sciences, University of South Australia, Adelaide, SA, Australia; ^3^Department of Cardiothoracic Surgery, Affiliated Hospital, Southwest Medical University, Luzhou, China

**Keywords:** postoperative cognitive dysfunction, lncRNA, mRNA, Gene Ontology, genetic dysregulation, microarray analysis, bioinformatics analysis

## Abstract

Postoperative cognitive dysfunction (POCD) is serious disorder in the central nervous system common in aged patients after anesthesia. Although its clinical symptoms are well recognized, however, the molecular etiology of the POCD remains unrevealed. Similarly, neither gold standard molecular diagnosis nor effective treatment is available for POCD until the present. Therefore, we aimed to explore the molecular mechanism of this disorder through investigating lncRNAs and mRNAs associated with POCD human patients and investigate their underlying regulatory pathways. In this study, we recruited 200 patients requiring hip or knee replacement surgery. Their neurological functions were assessed at two time points, 1 day before the surgery and 30 days post-surgery. In parallel, serum samples were collected from the participants to analyze lncRNAs and mRNAs differential expression profile between POCD and non-POCD patients using microarray analysis. To further investigate the role differentially expressed mRNA and lncRNAs, Gene Ontology (GO), pathway analyses on mRNAs and lncRNA-mRNA interaction network were performed. As a result, 68 lncRNAs and 115 mRNAs were dysregulated in the POCD group compared to non-POCD group. Among them, the top 10 upregulated lncRNAs and 10 downregulated lncRNAs were listed for enrichment analysis. Interestingly, we found that these lncRNA and mRNA are involved in biological process, molecular function, and cellular component in addition to various signaling pathways, suggesting that the pathogenesis of POCD involves lncRNAs and mRNAs differential expression. Consequently, the genetic dysregulation between the non-POCD and POCD patients participates in the occurrence and development of POCD, and could be served as diagnostic biomarkers and drug targets for POCD treatment.

## Introduction

Postoperative cognitive dysfunction (POCD) is a severe central nervous system (CNS) complication in the cognitive function common in elderly patients after anesthesia and surgery. The condition involves symptoms rang from mild to serious cognitive deficits affecting life quality (Hartholt et al., 2012; Arora et al., 2014). POCD is clinically characterized by different levels of memory lost, emotional disorder such as anxiety, confusion, and depression, and in extreme cases, coma and death (Bekker and Weeks, 2003). While the neuropsychological test for POCD typically includes Mini-Mental State Examination (MMSE), Wechsler Adult Memory Scale (WMS), and Minnesota Multiphasic Personality Inventory (MMPI; Moller et al., 1998; Krenk et al., 2010; Monk and Price, 2011). Owing to differences in definitions and testing methods, the incidence of POCD has been reported globally with high prevalence. Generally, the incidence of POCD was estimated up to 26% at 7 days and 10% at 3 months in beyond than 65 years old patients (van Harten et al., 2012; Silbert et al., 2014). Disease pathogenicity is complex and multifactorial. It includes central cholinergic system degradation, central inflammation (Hu et al., 2014), tau phosphorylation (Planel et al., 2007), β-amyloid deposition (Xu et al., 2012), and neuronal apoptosis (Vacas et al., 2014). Although POCD occurs in high prevalence and has severe effect on the affected patients (Witlox et al., 2010). However, there is no standard treatment of the disease or measures that can be taken to prevent or reduce the incidence of POCD (Pappa et al., 2017).

Long non-coding RNAs (lncRNAs) are set of non-coding RNA with more than 200 nucleotides lengths (Marchese et al., 2017; Muret et al., 2017). Interestingly, increasing number of publications presented evidences of critical role in various biological processes, mainly on chromosome dosage compensation, genomic imprinting, target mimicry, and functional protein trafficking (Clark and Mattick, 2011; Nagano and Fraser, 2011; Wang and Chang, 2011). In addition, lncRNAs involve in various biological function, such as cell proliferation, differentiation, apoptosis, and regulating the expression of target genes during transcription, post-transcription, and epigenetic levels (Yoon et al., 2013). At the present, researchers mainly relay on computational and mathematical methods to predict lncRNA functional domains at structural levels. Among them, chromatin isolation by RNA purification (Ch IRP) and RNA-binding protein immunoprecipitation (RIP) have widely used to investigate the interaction between lncRNA with genes and proteins respectively (Yoon et al., 2013). Moreover, lncRNAs have been confirmed to associate with number of neurological disorders in addition to many other diseases like cancer and immune diseases (Ponting et al., 2009; Ulitsky and Bartel, 2013; Maass et al., 2014; Isin and Dalay, 2015). Nevertheless, the relation of lncRNAs and POCD is unclear and needs to be explored. Therefore, this study is to investigate the role of lncRNA and mRNA in the development of POCD.

We selected hip and knee replacement patients as clinical cases to detect the lncRNAs and mRNAs profiles in POCD patients non-POCD patients using microarray analysis. Furthermore, Gene Ontology (GO) and KEGG analyses were performed to study the potential functions of the dysregulated lncRNAs and mRNA. Finally, network interaction of lncRNAs–mRNAs in the POCD patients was constructed.

## Materials and Methods

### Ethical Considerations and Study Approval

The study was approved by the ethic committee of Affiliated Traditional Chinese Medicine Hospital of Southwest Medical University (project number KY2018001) and registered with Chinese Ethics Committee of Registering Clinical Trials in 2018 (Unique identifier ChiCTR-INR-15007607). All participating patients were informed with the aim of the study and signed the related consent forms.

### Selection of Patients

Two hundred patients admitted to Affiliated Traditional Chinese Medicine Hospital of Southwest Medical University to carry out hip or knee replacement surgery have participated in this study. Patients selected based on firm criteria which were (1) age at >60 years old; (2) anesthetic procedure (American Society of Anesthesiologists Grade 1–3); and (3) mental health. It was decided to exclude patients beyond 85 years old, patients with nervous system diseases, preoperative stoke, and other central nerve system diseases cases (preoperative MMSE < 24 scores), and patients who suffered from multiple injuries and craniocerebral injury.

### Anesthesia and Surgery

All patients were asked to fast from food 8 h before the operation and from water 3 h before anesthesia. The anesthetic procedure was combined of spinal and epidural anesthesia at the L3–L4 intervertebral disc in a side-lying position using an 18-gage needle and 25-gage spinal needle (Tuo Ren, Henan, China). Then, the 2 ml mixed solution consisting of 0.5% bupivacaine (Hua Lu, Shandong, China) and 3.33% glucose (Shuang He, Beijing, China) was slowly injected to subarachnoid space. The vital sign of patients was monitored during the surgery. All the participants were given the same analgesia doses 12 μg/kg fentanyl (Ren Fu, Yichang, China) and 10 mg tropisetron hydrochloride (Ren Fu, Yichang, China) for 2 days.

### Neuropsychological Assessment

For cognitive function evaluation, a battery of neuropsychological tests were followed 1 day before and 1 month after the surgery by trained physician. The tests consisted of the MMSE, Symbol Digit Modalities Test (SDMT), Digit Span Test (DST), Trail Making Test A, and Clock Drawing Test (CDT). The cognitive functions were principally assessed including memory, learning concentration, and executive function. Briefly, MMSE was designed to evaluate and quantify the global cognitive state, assess memory, spatial–temporal orientation, and reasoning (Bertolucci et al., 1994). SDMT was used to test psychomotor speed and attentional control (Valentin et al., 2016). DST is a subtest of WAIS-RC, which instructed participants to memorize numbers, and repeat them reversely and was used for memory assessment (Gong, 1992). Trail Making Test A was adopted to assess executive function, selective and alternating attention (Valentin et al., 2016). While CDT was applied for visual space function, concentration, the planning, and execution function of the action assessment (Aprahamian et al., 2010).

A group 30 health volunteers were also assessed as normal reference of cognitive functions and were corresponding with their patient’s counterparts based on education level, age, and gender. The cognitive functions were evaluated twice in 7 days intervals. Patients were separated into POCD and non-POCD groups. The (SD) value, mean variation, and baseline score were calculated to statistically validate the data (Rasmussen et al., 2001). The learning effect and baseline score were deducted from the score of neuropsychological tests (Duan et al., 2018) and, divided by the SD of the baseline score of the healthy group. This result was defined as *Z* score, and the participants *Z* ≥ 1.96 were included in POCD (Silbert et al., 2014).

### Microarray Analysis

Three days after the surgery, 8 ml of peripheral blood samples were collected from the patients in pro-coagulant tube and centrifuged (at speed of 2500 rpm for 10 min). The supernatant was taken and stored in liquid nitrogen until the test. Random number table was applied to select three patients with early POCD (labeled A, B, C) and three patients with non-POCD was as control (labeled D, E, F). Total RNA was extracted using TRIzol reagent kit based on vendor instructions, the plasma. RNA integrity was assessed by Agilent Bioanalyzer 2100 (Agilent Technologies), and the purity was measured with NanoDrop2000 spectrophotometer (Thermo Scientific). RNA samples with OD260/280 > 1.8 and OD260/230 > 1.5 were taken for further testing.

RNA samples were sent to Shanghai OE Biotech Inc. (Shanghai, China) to carry out. The sample labeling, hybridization, and washing were performed according to service provider protocols. In brief, cDNAs was synthesized for from RNA samples then synthesized cRNAs. Second cycle cDNAs were synthesized from cRNAs and followed by fragmentation and biotin labeling, the second cycle cDNAs were hybridized onto the Affymetrix Human OE lncRNA microarray (Affymetrix, Santa Clara, CA, United States), comprising 66,741 human lncRNAs and 25,986 coding mRNA. The chips were then washed, stained, and scanned by Affymetrix Scanner 3000 (Affymetrix, Santa Clara, CA, United States).

### Bioinformatics Analysis

Affymetrix Gene Chip Command Console (version 4.0, Affymetrix) software was used to extract raw data. Expression Console (version1.3.1, Affymetrix) software offered RMA normalization for both gene and exon level analysis. Then, Genesrping software (version 13.1; Agilent Technologies) was employed to carry out the basic analysis. Venn Diagram software was performed to generate high-resolution Venn plots.

**FIGURE 1:**
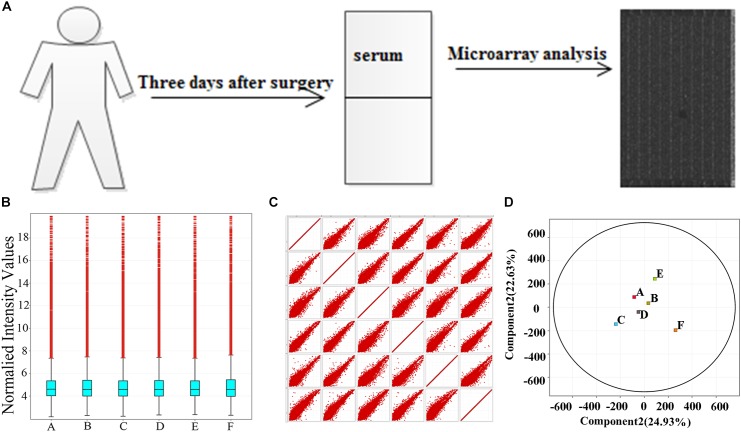
Samples self-test. **(A)** The workflow of the study. **(B)** Box–whisker plot shows the dispersion. **(C)** Scatter plot shows the total trend of dispersion. **(D)** PAC shows the rationality of POCD group (labeled A, B, C) and non-POCD group (labeled D, E, F). POCD, postoperative cognitive dysfunction. A, B, and C in the images represent three samples in POCD group, and D, E, and F represent three samples in non-POCD group.

To understand the function of lncRNAs associated with the PODC, we first established the role of differentially expressed mRNA through GO and KEGG analyses. The lncRNAs then were linked to their adjacent mRNAs to establish their potential function (Zhao et al., 2015). Pearson correlation and *p*-values were calculated and followed to measure pathway correlation to the conditions. Method of Storey (2002) was used to identify the false discovery rate. Venn diagram was applied to display the co-regulated mRNAs of lncRNAs. GO analysis was used for further insight on the biological function and molecular mechanism of the dysregulated genes. The workflow of the study is described in **Figure 1A**.

### Statistical Analysis

In this study, SPSS software version 17.0 (SPSS, Inc., Chicago, IL, United States) and GraphPad Prism 5.0 (GraphPad Software, Inc., La Jolla, CA, United States) were used for data analysis and the experimental results were presented as mean values ± standard deviation (SD); *P* < 0.05 was viewed as statistically significant difference.

## Results

### Characteristics and Neuropsychological Test of Participates

Of these 200 patients, 181 patients (91.5%) achieved the whole tests, while 19 patients (9.5%) did not complete the tests due personal reasons. The general characteristics of the patients including age, body mass index, gender, and education are presented in **Table 1**. No significance variations were noticed in the scores of the all participants at first day before surgery (*P* > 0.05). On the other hand, CDT scores were noticeably lower in some patients and they were taken as POCD group compared with non-POCD group after 1 month of the surgery (**Table 2**). Whereas, the general characteristics and intraoperative conditions were statistically insignificant. One month after hip or knee replacement surgery, 21 patients (11.6%) were diagnosed as cognitive dysfunction, performed remarkably worse verbal IQ, performance IQ, and working memory compared with other non-POCD patients (**Table 3**).

**Table 1:** General characteristics and cognitive functions of participants from study and control groups at baseline

Item (baseline)	Study group (*n* = 181)	Control group (*n* = 30)	*p-*value
Age (years)	77.1 ± 2.5	77.3 ± 3.1	0.696
Weight (kg)	63.8 ± 10.7	65. 7 ± 12.4	0.380
Education (years)	8.3 ± 2.4	9.1 ± 2.6	0.096
MMSE scores	28.2 ± 1.6	28.8 ± 2.1	0.071
DST scores	16.3 ± 1.8	16.6 ± 1.6	0.392
CDT scores	2.5 ± 0.5	2.6 ± 0.6	0.326
SDMT scores	31.3 ± 3.2	30.4 ± 3.5	0.161
TMT-A scores	41.1 ± 5.8	42.4 ± 6.6	0.266


MMSE, Mini-Mental State Examination; DST, Digit Span Test; CDT, Clock Drawing Test; SDMT, Symbol Digit Modalities Test; TMT-A, Trail Making Test A.

**Table 2:** General characteristics, intraoperative indicators and cognitive functions of patients from non-POCD and POCD at baseline.

Item (baseline)	Non-POCD (*n* = 160)	POCD (*n* = 21)	*p*-value
Age (years)	76.8 ± 2.7	77.2 ± 3.1	0.531
Weight (kg)	63.4 ± 11.2	64 ± 12.4	0.820
Education (years)	8.3 ± 1.4	8.6 ± 2.6	0.357
ASA classification (I/II/III)	32/104/24	5/14/2	0.501
Operation time (min)	84.9 ± 18.8	79.8 ± 19.6	0.246
Intraoperative blood loss (ml)	296.4 ± 37.2	290.6 ± 43.8	0.512
Intraoperative urine output (ml)	376.3 ± 86.2	370.4 ± 84.5	0.768
Intraoperative blood sugar (mmol/l)	5.71 ± 0.58	5.78 ± 0.66	0.610
MMSE scores	28.0 ± 1.4	28.4 ± 1.8	0.236
DST scores	16.1 ± 1.4	16.4 ± 1.5	0.361
CDT scores	2.4 ± 0.5	2.7 ± 0.6	0.013
SDMT scores	31.0 ± 3.6	31.6 ± 3.9	0.478
TMT-A scores	40.1 ± 7.8	42.3 ± 8.6	0.231


*POCD, postoperative cognitive dysfunction; MMSE, Mini-Mental State Examination; DST, Digit Span Test; CDT, Clock Drawing Test; SDMT, Symbol Digit Modalities Test; TMT-A, Trail Making Test A.*

**Table 3:** Neuropsychological test scores of non-POCD group and POCD group.

Item	Non-POCD (*n* = 160)	POCD (*n* = 21)	*p-*value
MMSE scores	27.2 ± 0.6	25.9 ± 1.1	0.000
DST scores	14.1 ± 1.6	13.1 ± 1.1	0.006
CDT scores	2.3 ± 0.5	1.9 ± 0.7	0.001
SDMT scores	31.3 ± 3.2	28.9 ± 3.5	0.002
TMT-A scores	41.4 ± 5.1	38.7 ± 6.1	0.027


*POCD, postoperative cognitive dysfunction; MMSE, Mini-Mental State Examination; DST, Digit Span Test; CDT, Clock Drawing Test; SDMT, Symbol Digit Modalities Test; TMT-A, Trail Making Test A.*

### Samples Quality Detection

Chip data dispersion was illustrated in box–whisker plot, scatter plot, and PCA. From box–whisker plot, no statistical variance was seen neither in the lncRNAs distributions nor in mRNAs expression profile in the samples (**Figure 1B**). As shown in **Figures 1C,D**, the scatter plot and PCA were applied to measure dissimilarities in lncRNAs expression between POCD group and control group, the detection revealed that in scatter plot and PCA, the overall distribution of the two groups of data is concentrated (**Figures 1C,D**).

### Differential Expression Profile of lncRNAs

**FIGURE 2:**
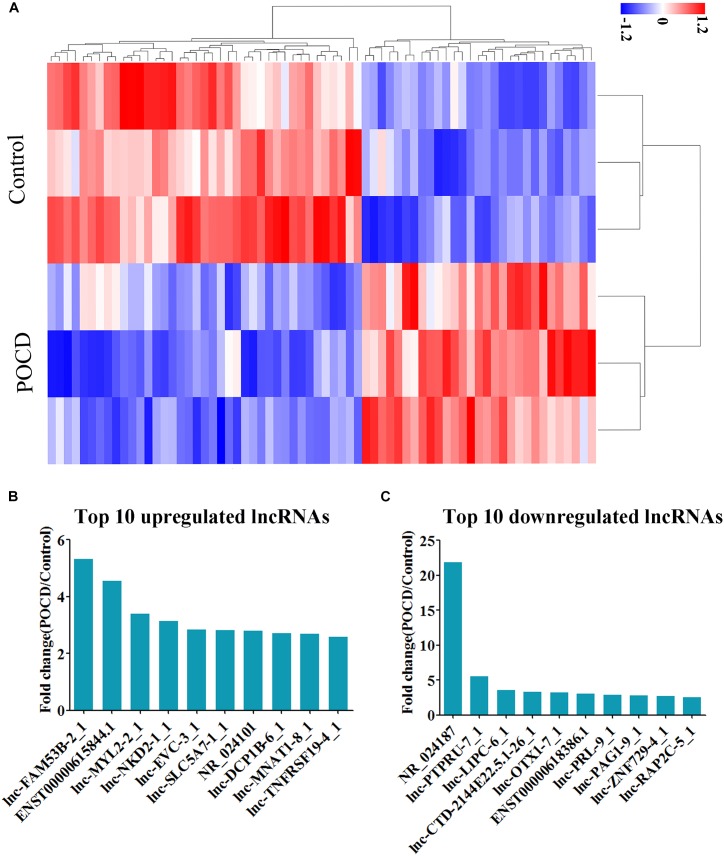
Differential expression of lncRNAs. **(A)** Heatmap of lncRNAs in POCD group vs. non-POCD group. **(B)** The top 10 upregulated lncRNAs. **(C)** The top 10 downregulated lncRNAs. POCD, postoperative cognitive dysfunction.

Hierarchical clustering analysis data and heatmap (**Figure 2A**) have shown the distinct lncRNAs expression profile between the groups; 68 lncRNAs were observed to be differentially expressed in the POCD patients, including 29 and 39 upregulated and downregulated, respectively (see **Supplementary Tables S1, S2**). The top 10 upregulated lncRNAs were identified as lnc-FAM53B-2_1, ENST00000615844.1, lnc-MYL2-2_1, lnc-NKD2-1_1, lnc-EVC-3_1, lnc-SLC5A7-1_1, NR_024101, lnc-DCP1B-6_1, lnc-MNAT1-8_1, and lnc-TNFRSF19-4_1 (**Figure 2B**). Whereas the top 10 downregulated lncRNAs were NR_024187, lnc-PTPRU-7_1, lnc-LIPC-6_1, lnc-CTD-2144E22.5.1-26_1, lnc-OTX1-7_1, ENST00000618386.1, lnc-PRL-9_1, lnc-PAG1-9_1, lnc-ZNF729-4_1, and lnc-RAP2C-5_1 (**Figure 2C**). These lncRNAs were further studied by bioinformatics tools.

### Bioinformatics Analysis of Upregulated mRNAs in POCD Group

**FIGURE 3:**
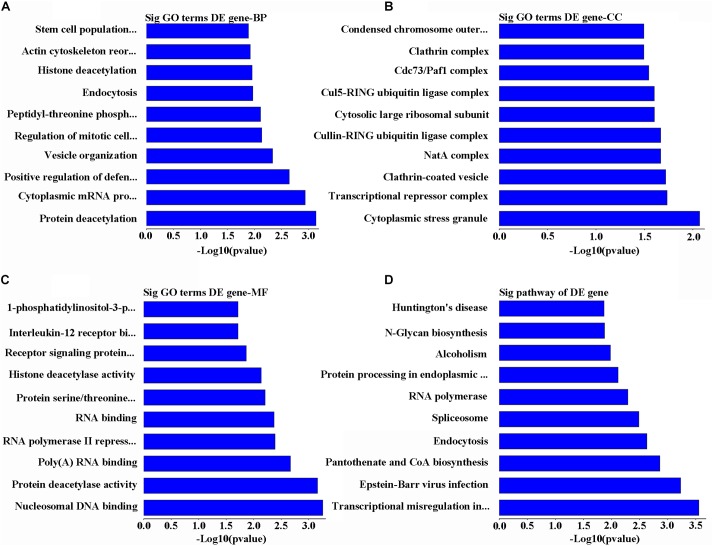
Analysis of bioinformatics for the upregulated genes. **(A)** The map of the top 10 biological process. **(B)** The map of the top 10 cell components. **(C)** The map of the top 10 molecular functions. **(D)** The top 10 significant pathways of upregulated genes in POCD group.

Gene Ontology analysis for the main overexpressed mRNA has revealed that these transcriptomes are involved in biological processes through series of functions including deacetylation, cytoplasmic mRNA processing, stem cell population maintenance, and positive regulation of immunity against viruses by vesicle organization, regulation of mitotic cell cycle, peptidyl-threonine phosphorylation, endocytosis, histone deacetylation, and actin cytoskeleton reorganization (**Figure 3A**). At the same time, these mRNA sequences were predicted to target different cellular components including cytoplasmic stress granule, transcriptional repressor complex, clathrin-coated vesicle, NatA complex, cullin-RING ubiquitin ligase complex, cytosolic large ribosomal subunit, Cul5-RING ubiquitin ligase complex, Cdc73/Paf1 complex, clathrin complex, condensed chromosome, and outer kinetochore (**Figure 3B**). On the molecular level, the other upregulated RNAs were involved in modulating the cells by being nucleosomal DNA binding, RNA binding factors, protein deacetylase activity, poly(A), RNA polymerase II repressing factor, serine/threonine kinase, signaling receptors, interleukin-12 receptor binding factor, and 1-phosphatidylinositol-3-phosphate 4-kinase activity (**Figure 3C**). On other dimension, KEGG data of the upregulated mRNAs were predicted to take part in transcriptional misregulation in cancer, Epstein–Barr virus infection, pantothenate and CoA biosynthesis, endocytosis, spliceosome, RNA polymerase, protein processing in endoplasmic reticulum, alcoholism, N-glycan biosynthesis, and Huntington’s disease (**Figure 3D**).

### Bioinformatics Analysis of Downregulated mRNAs in POCD Group

**FIGURE 4:**
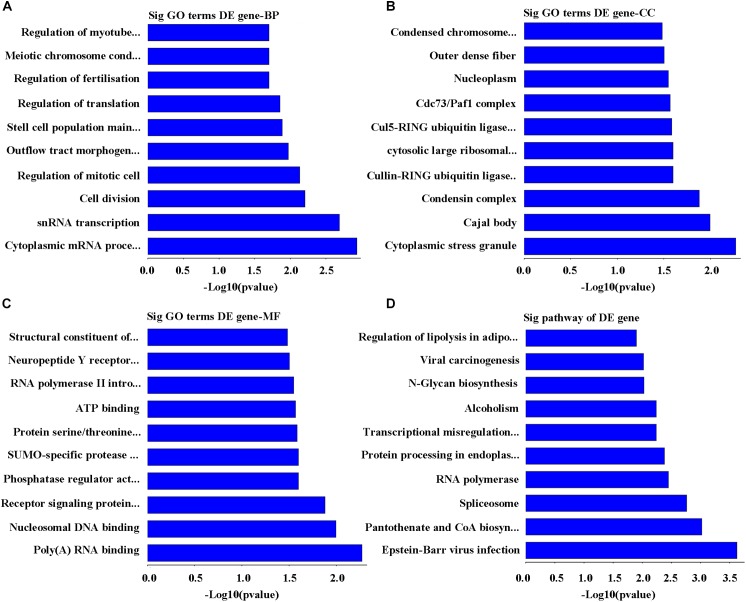
Analysis of bioinformatics for the downregulated genes. **(A)** The map of the top 10 biological process. **(B)** The map of the top 10 cell components. **(C)** The map of the top 10 molecular functions. **(D)** The top 10 significant pathways of downregulated genes in POCD group.

On the basis of the enrichment scores, the top 10 biological processes targeted by downregulated mRNAs were identified as cytoplasmic mRNA processing body assembly, snRNA transcription, polymerase II promoter, cell division, regulation of mitotic cell cycle, outflow tract morphogenesis, stem cell population maintenance, regulation of translation, regulation of fertilization, meiotic chromosome condensation, and regulation of myotube differentiation (**Figure 4A**). Whereas the highest 10 cellular components related to the downregulated mRNAs were cytoplasmic stress granule, Cajal body, condensin complex, cullin-RING ubiquitin ligase complex, cytosolic large ribosomal subunit, Cul5-RING ubiquitin ligase complex, Cdc73/Paf1 complex, nucleoplasm, outer dense fiber, and condensed chromosome outer kinetochore (**Figure 4B**). Similarly, the top 10 functions on molecular mechanism include poly(A) RNA binding, nucleosomal DNA binding, signaling protein receptor, serine/threonine kinase, phosphatase regulator, SUMO-specific protease activity, protein serine/threonine kinase activity, ATP binding, RNA polymerase II intronic transcription regulator, neuropeptide Y receptor activity, and structural constituent of ribosome (**Figure 4C**). Laterally, the top 10 affected pathways of mRNA sequences downregulated were Epstein–Barr virus infection, pantothenate and CoA biosynthesis, spliceosome, RNA polymerase, Protein processing in endoplasmic reticulum, transcriptional misregulation in cancer, alcoholism, N-Glycan biosynthesis, carcinogenesis viral RNA, and adipocytes lipolysis regulation (**Figure 4D**).

### Co-expressed Analysis of lncRNA and mRNA in POCD Group

**FIGURE 5:**
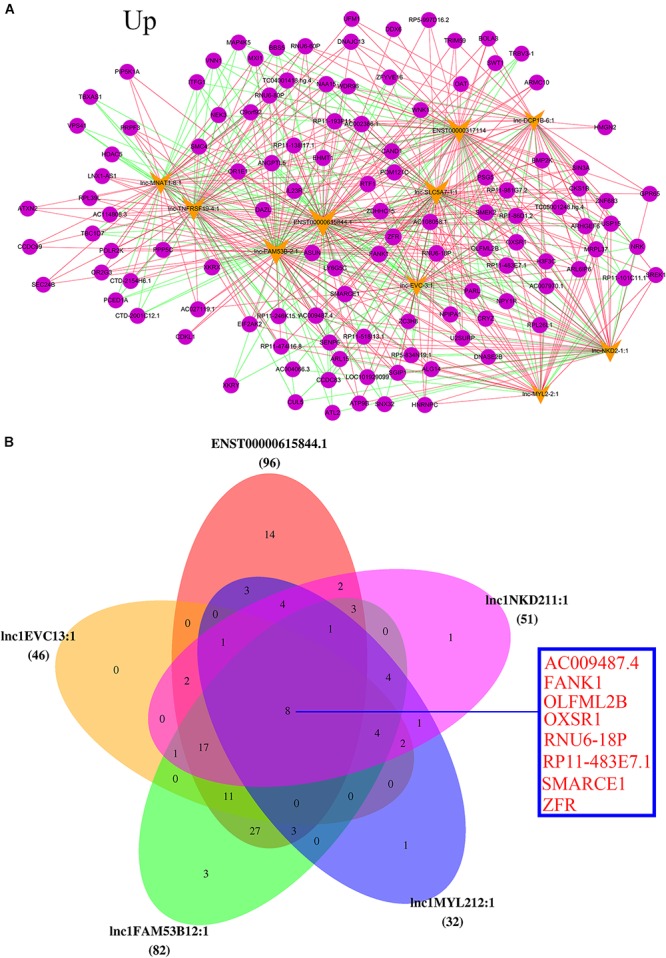
Analysis of upregulated lncRNAs-mRNA co-expression. **(A)** The top 10 upregulated of regulatory network. Blue nodes represent lncRNAs and yellow nodes represent target genes. The pink lines represent positive correlations and the green lines represent negative correlations. **(B)** Top five upregulated lncRNAs co-regulated mRNA.

Based on the information of *trans* (trans-prediction) and Venn analyses, the lncRNA–mRNA interaction network was constructed. The data showed that the 10 upregulated lncRNAs were correlated with amounts of mRNAs through *trans* analysis. The lnc-FAM53B-2_1 in particular was the highest upregulated lncRNA. Interestingly, it interacted with the majority of differentially dysregulated mRNA sequences. It was also noticed that SMARCE1, ATXN2, SMC4, and IL23R were remarkably unregulated in POCD patients (**Figure 5A**). Venn diagram showed that the highest five upregulated lncRNAs were lnc-FAM53B-2_1, ENST00000615844.1, lnc-MYL2-2_1, lnc-NKD2-1_1, and lnc-EVC-3_1. While the main overexpressed mRNAs were AC009487.4, FANK1, OLFML2B, OXSR1, RNU6-18P, RP11-483E7.1, SMARCE1, and ZFR (**Figure 5B**).

**FIGURE 6:**
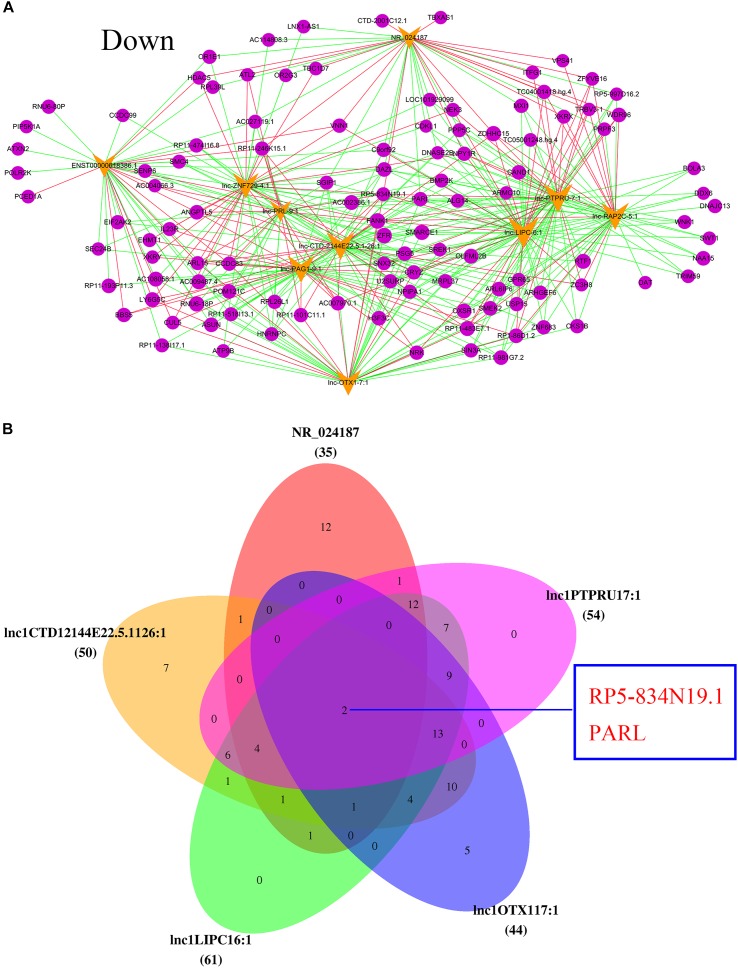
Analysis of downregulated lncRNAs-mRNA co-expression. **(A)** The top 10 downregulated of regulatory network. Blue nodes represent lncRNAs and yellow nodes represent target genes. The pink lines represent positive correlations and the green lines represent negative correlations. **(B)** Top five downregulated lncRNAs co-regulated mRNA.

In addition, the top 10 downregulated lncRNAs were also associated with many mRNA sequences. As the most downregulated lncRNA in POCD, NR_024187 was correlated with that of NEK3, XKRX, VNN1, TBXAS1, and HDAC5 (**Figure 6A**). Venn diagram also showed that the main five downregulated lncRNAs, were NR_024187, lnc-PTPRU-7_1, lnc-LIPC-6_1, lnc-CTD-2144E22.5.1-26_1, and lnc-OTX1-7_1 and they were slightly correlated with RP5-834N19.1 and PARL mRNA (**Figure 6B**).

### Differential Expression of mRNAs

**FIGURE 7:**
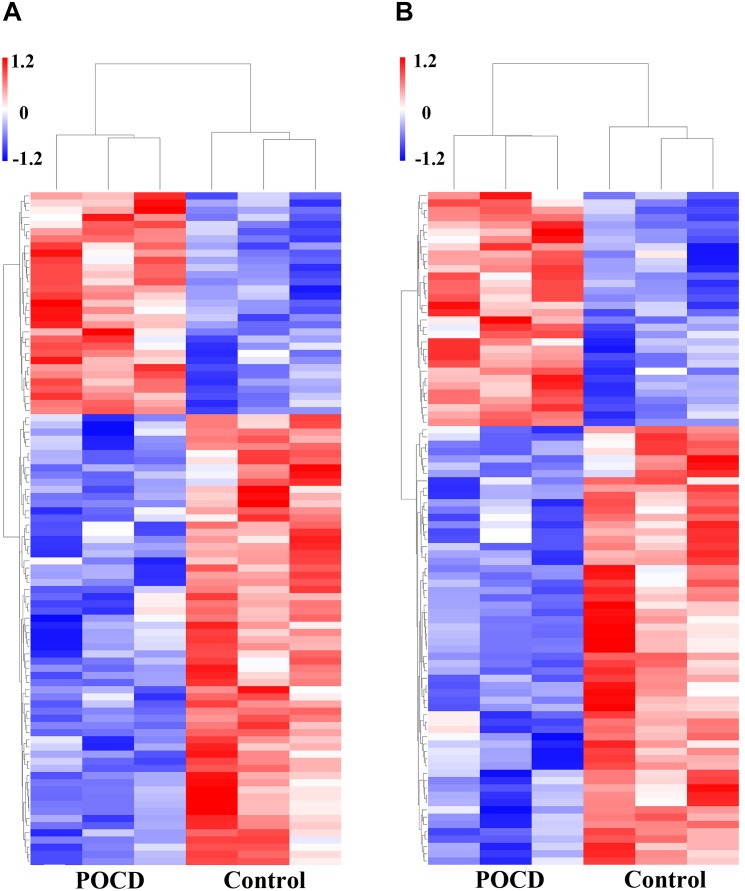
Differential expression of mRNAs. Heatmaps of mRNAs in POCD patients vs. non-POCD patients. **(A)** The top 10 of upregulated mRNAs. **(B)** The top 10 of downregulated mRNAs.

Microarray data analysis also showed the overexpressed genes. Total of 115 mRNA sequences were dysregulated between the POCD and control groups (FC > 2, *P* < 0.05). Among them, 39 mRNAs were upregulated and 76 were downregulated (*P* < 0.05). Variations in mRNA expression levels of the POCD and non-POCD group are illustrated in **Figure 7**.

## Discussion

Postoperative cognitive dysfunction is a postoperative complication with high incidence and poor prognosis (van Dijk et al., 2007). At present, the mechanism of POCD is focused on inflammation, but there is little report on lncRNA level while the specific molecular mechanism is not completely understood (Terrando et al., 2011; Zhang X. et al., 2016).

As a class of important ncRNA, lncRNA plays vital role in central system (Kong et al., 2017; Liu et al., 2018). In recent years, several reports suggested that lncRNA is involved in neural physiology and diseases like Alzheimer’s disease (AD) and Parkinson’s disease (PD; Wang et al., 2018). Side by side with the identification of numerous biomarkers linked with the POCD, for instance, Aβ and tau (Wu et al., 2018), pro-inflammatory protein S100A8 (Lu et al., 2015), and preoperative levels of immunoglobulin M (Mathew et al., 2003). Along with the previously reported, NF-kappa B, IL-1b, and TNF-α (Renard et al., 1997; Chandel et al., 2000). Meanwhile, the growing number of evidences have demonstrated the crucial role of lncRNAs in neurotransmission, memory structure, and synaptic plasticity in the brain (Wang et al., 2018), such as the newly discovered lncRNA, MALAT1 (Shang et al., 2018), NEAT1 (Yan et al., 2018), and H19 (Zhao et al., 2017). In a similar trend, lncRNAs may present biomarker for molecular diagnosis of diseases. Recent evidence has shown that lncRNAs exhibit high percentage of the total transcriptome (Guennewig and Cooper, 2014; Briggs et al., 2015; Chen et al., 2016) and strongly related to neural differentiation and synaptic plasticity (Briggs et al., 2015; Gudenas and Wang, 2015). In the meantime, many researchers have reported considerable progress toward thorough understanding of the relationship between lncRNAs and brain physiology and diseases (Gudenas and Wang, 2015; Chen et al., 2016).

In the present study, various neuropsychological tests were adopted to evaluate cognitive functions 1 day before and 30 days after knee or hip replacement surgery to distinguish between POCD and non-POCD patients. Then, lncRNA sequences were studied from all the patients using developed microarray chips. Microarray analyses revealed that there were total of 29 upregulated lncRNAs and 39 mRNAs, 39 downregulated lncRNAs, and 76 mRNAs associated with POCD. Likewise, the GO and KEGG were performed to predict the roles of the dysregulated lncRNAs, and the co-expression mRNA. Furthermore, network interaction of lncRNAs and mRNAs in the POCD patients was constructed to explore the relationship between lncRNAs and mRNAs with the disease. As POCD symptoms are memory decline, poor attention, learning disability, and deficit function execution. Therefore, this experiment uses a series of neuropsychology tests to assess these five categories of cognition functions to distinguish whether cognitive impairment existed before surgery it has occurred after the surgery. To increase the accuracy, all cognitive functions were measured by more than one test to diagnose the POCD patients.

### Microarray Analysis

Wei et al. (2017) were the first to report lncRNA linkage to POCD in animal model. Whereas, our study has discovered various lncRNAs and mRNAs associated with POCD human patients and have expanded the understanding of the role of non-coding RNA in POCD. Microarray was used to investigate differentially expressed lncRNAs and mRNAs which then become the main data for the subsequent bioinformatics analyses of the study. This working strategy has been applied and validate in studying many diseases including cancer (Han et al., 2018; Liang et al., 2018; Lou et al., 2018), cerebral ischemia-induced lung injury (Hu et al., 2017), and traumatic brain injury (Lyu et al., 2017). In our study, microarray was used to study the changes in expression of lncRNAs and mRNAs in POCD group. The results presented 68 differentially expressed lncRNAs and 115 mRNAs in POCD group compared to non-POCD group. lnc-FAM53B-2_1 and NR_024187 were the most obvious expressions of upregulated and downregulated lncRNA, respectively. This has confirmed the previous animal model study in showing the correlation of POCD with changing levels of lncRNAs and mRNAs expression. Microarray analysis combining with GO and KEGG analyses provide reliable platforms for subsequent study for further understanding for the occurrence and progression of POCD on molecular levels.

### Bioinformatics Analyses

Bioinformatics analyses were aimed to indicate the potential functions of the dysregulated lncRNAs that have been identified in our study. GO analysis has illuminated the functions of genes through biological process, cellular component, and molecular function. While KEGG is normally used to study the possible roles of expressed RNA with correlation to cell signaling pathways (Hu et al., 2017).

In our analysis of biological process, it was noticed that the differentially expressed lncRNAs, mainly participated in process of protein and histone deacetylation, cytoplasmic mRNA processing body assembly, regulation of cell cycle, and stem cell population. Previous study has also pointed out the role of histone acetylation/deacetylation in neurodegenerative conditions (Verma et al., 2017). In concur with other recent works which presented evidences of histone deacetylase-2 as player in stress-induced cognitive impairment via histone deacetylation and PI3K/AKT pathway modulation (Verma et al., 2017; Wu et al., 2017), our results are consent with these published studies. However, the recognized biological processes leading to the pathogenesis of POCD, such as inflammation, stress, and apoptosis (Tian et al., 2015; Johansson et al., 2017; Zhang et al., 2018), are less prominent in our results. Sirtuin1 (SIRT1) is a deacetylase protein that reported to mediate hippocampal neuronal apoptosis in mice and causing cognitive deficit (Min et al., 2018). Our results have presented histone deacetylase’s critical role in the pathogenesis of POCD. In addition, molecular function analysis has shown that protein deacetylase activity, poly (A) RNA binding, interleukin-12 receptor binding, and serine/threonine kinase were also involved in the POCD. Histone deacetylase is translated from HDAC gene, which is regulated by lncRNAs that was identified as differentially transcripted RNA in our study, indicating that lncRNAs exert their functions through regulating histone deacetylase activity in POCD pathogenesis. Yi et al. (2015) reported the close relatedness of inflammatory response with the changes in cognitive function in cardiopulmonary bypass patients (Yi et al., 2015). In a similar manner, interleukin-12 and its receptor were demonstrated to influence cognitive function (Johansson et al., 2017; Vonder Haar et al., 2017; Wu et al., 2017). Likewise, cytokines and cytokines receptor participate apoptosis and immune regulation through Jak-STAT signaling pathway by specifically activate STAT4 (Darnell et al., 1994; Kang and Kang, 2008). In regard to the observation of the role serine/threonine kinase in the POCD, similar observation was reported diabetic related cognitive impairment. As the expression of phosphorylated serine/threonine kinase (p-AKT) increased under in those patients, reflecting the associated of serine/threonine kinase in cognitive impair function (Zhou et al., 2017).

Furthermore, pathway analysis revealed that there were 10 pathways implicated in POCD patients, Epstein–Barr virus infection, and pantothenate and CoA biosynthesis were the highly enriched pathways in POCD patients. Epstein–Barr virus, a human gamma-herpesvirus, potentially facilitates the damage of autoimmunity and CNS tissue, causing psychiatric symptoms and cognitive dysfunction (Wynne et al., 2010). There are evidences to demonstrate critical function of pantothenate kinase 2 (pank2) and CoA homeostasis in neuronal development and functioning in zebra fish (Zizioli et al., 2016). Our data have revealed the elevated level of enrichment of pantothenate and CoA biosynthesis in POCD group, suggesting the linkage of this pathway to POCD pathogenesis. The other moderately enriched-pathways like Jak-STAT signaling pathway, MAPK signaling pathway, and metabolic pathways in our study were also recorded to be involved in occurrence and development of POCD in rats’ model (Wei et al., 2017). Inflammatory reaction can trigger several metabolic pathways, which are closely related to the changes in cognitive function in patients undergoing cardiopulmonary bypass (Yi et al., 2015). It has been confirmed the phosphoinositide 3-kinase (PI3K)/AKT/glycogen synthase kinase-3β (GSK-3β) pathway are induced due to isoflurane anesthesia induced-inflammation, stress, and apoptosis, thus resulting to cognitive impairment (Zhang Y. et al., 2016). These studies combined with ours can provide a comprehensive insight of the mechanism of POCD.

### Co-expression of lncRNA and mRNA

Until the present, the functions of lncRNAs are not entirely clear. The co-expression network of aberrantly expressed mRNAs and lncRNAs in POCD was constructed to further understand of the role of lncRNAs via calculation and inference.

In the present study, we have selected the top 10 up and downregulated lncRNAs to construct the lncRNA-mRNA co-expression network of interactions to predict the genes targeted by lncRNA. We found that the increased lncRNA, lnc-FAM53B-2_1, correlated with that of SMARCE1, ATXN2, SMC4, and IL23R, while main reduced lncRNA was NR_024187 and has influence on NEK3, XKRX, VNN1, TBXAS1, and HDAC5. Previous study showed SMARCE1 to be associated with the occurrence and development of non-syndromic intellectual disability (Santen et al., 2013). Increasing number of evidences found that ATXN2 gene plays key role in neural diseases like dementia and motor neuron disease which could affect cognitive function (van Blitterswijk et al., 2014). HDAC5 has been confirmed to be involved in cognitive function including learning and memory process (Taniguchi et al., 2017). Furthermore, we discovered that the top five upregulated lncRNAs commonly regulating eight mRNAs, two among them were the mainly affected mRNA sequences. These implied that POCD is a multigene induced disease and lncRNAs may participant in POCD via regulating mRNA expression. These genes are reported in POCD for the first time assuming they will serve as the base for further research in the future.

However, it will be worthwhile to evaluated neurological function in long-term manner in POCD patients. In addition, more hands on researches should be conducted to investigate the exact mechanisms and function of genes that have been reported in this study to examine the specific interaction mechanisms between lncRNAs and POCD.

## Conclusion

We have recorded that lncRNAs and mRNAs in serum were differentially expressed in patients with POCD compared with patients without POCD after hip or knee replacement surgery. These vital findings may contribute to well understand the mechanism of POCD and provide new biomarkers for diagnosis and monitoring the progression of POCD and potential therapeutic target for POCD treatment.

## Author Contributions

F-XY and YZ conceived and designed the experiments. YZ, Y-XL, Q-XX, and QL performed the experiments. Y-XL, RD, ID, and JB analyzed the data. YZ, MA-H, and Y-XL wrote the manuscript. MA-H and ID participated in improving the paper and making the language more authentic during the revision.

## Conflict of Interest Statement

The authors declare that the research was conducted in the absence of any commercial or financial relationships that could be construed as a potential conflict of interest.
